# Home-based transcranial direct current stimulation in dual active treatments for symptoms of depression and anxiety: A case series

**DOI:** 10.3389/fpsyt.2022.947435

**Published:** 2022-10-06

**Authors:** Mónica Sobral, Raquel Guiomar, Vera Martins, Ana Ganho-Ávila

**Affiliations:** ^1^Faculty of Psychology and Educational Sciences, Center for Research in Neuropsychology and Cognitive Behavioral Intervention, University of Coimbra, Coimbra, Portugal; ^2^Neuroncircuit—e.Stim Clínica de Saúde Mental, Coimbra, Portugal; ^3^Coimbra Hospital and University Centre, Coimbra, Portugal

**Keywords:** tDCS, home-based, Flow, anxiety, depression, case series

## Abstract

Transcranial direct current stimulation (tDCS) is a potential treatment strategy across some psychiatric conditions. However, there is high heterogeneity in tDCS efficacy as a stand-alone treatment. To increase its therapeutic potential, researchers have begun to explore the efficacy of combining tDCS with psychological and pharmacological interventions. The current case series details the effect of 6–10 weeks of self-administered tDCS paired with a behavioral therapy smartphone app (Flow™), on depressive and anxiety symptoms, in seven patients (26–51 years old; four female) presenting distinctive psychiatric disorders (major depression, dysthymia, illness anxiety disorder, obsessive-compulsive disorder, and anxiety disorders). tDCS protocol consisted of an acute phase of daily 30 min sessions, across 10 workdays (2 weeks Monday-to-Friday; Protocol 1) or 15 workdays (3 weeks Monday-to-Friday; Protocol 2). A maintenance phase followed, with twice-weekly sessions for 4 or 3 weeks, corresponding to 18 or 21 sessions in total (Protocol 1 or 2, respectively). The Flow tDCS device uses a 2 mA current intensity, targeting the bilateral dorsolateral prefrontal cortex. The Flow app offers virtually guided behavioral therapy courses to be completed during stimulation. We assessed depressive symptoms using MADRS-S and BDI-II, anxious symptoms using STAI-Trait, acceptability using ACCEPT-tDCS, and side effects using the Adverse Effects Questionnaire, at baseline and week 6 of treatment. Six patients underwent simultaneous cognitive-behavioral psychotherapy and two were on antidepressants and benzodiazepines. According to the Reliable Change Index (RCI), for depressive symptoms, we found clinically reliable improvement in five patients using MADRS-S (out of seven; RCI: −1.45, 80% CI; RCI: −2.17 to −4.82, 95% CI; percentage change: 37.9–66.7%) and in four patients using BDI-II (out of five; RCI: −3.61 to −6.70, 95% CI; percentage change: 57.1–100%). For anxiety symptoms, clinically reliable improvement was observed in five patients (out of six; RCI: −1.79, 90% CI; RCI: −2.55 to −8.64, 95% CI; percentage change: 12.3–46.4%). Stimulation was well-tolerated and accepted, with mild tingling sensation and scalp discomfort being the most common side effects. This case series highlights the applicability, acceptability, and promising results when combining home-based tDCS with psychotherapy and pharmacotherapy to manage depression and anxiety symptoms in clinical practice.

## Introduction

Anxiety and mood disorders are amongst the most widespread psychiatric diseases, with a lifetime prevalence of 28.8 and 20.8%, respectively (1). Several pharmacological and psychological approaches are currently available. However, a high number of patients are classified as partial, non-responders or do not experience long-term clinical benefits (2, 3).

Transcranial direct current stimulation (tDCS) is an alternative and complementary therapeutic option, particularly promising due to its low cost, potential cost-effectiveness, easy application, and safe and tolerable profile (4, 5). As a non-invasive and non-pharmacological technique, tDCS applies a weak direct current through scalp electrodes (anode and cathode), modifying neuronal excitability and cortical activity according to stimulation parameters (6, 7). Stand-alone tDCS has already shown therapeutic efficacy in patients diagnosed with major depressive disorder (MDD) and anxiety (5, 7–9), being superior to sham in what concerns clinical response; however, its results are still highly heterogeneous (4, 10). In MDD, the hypoactive anode is usually positioned over the left dorsolateral prefrontal cortex (DLPFC) and the cathode over the right DLPFC or the right supraorbital or frontotemporal area (7).

To improve the therapeutic efficacy of tDCS and psychological interventions, researchers have been exploring the combination of both approaches. Using the Flow solution (a home-based tDCS and app-based psychological intervention; Flow Neuroscience™, Malmö, Sweden; https://flowneuroscience.com/), Borrione et al. (11) found that four out of five patients with MDD responded substantially to the treatment, suggesting a synergistic/additive effect. Furthermore, promising effects have been reported for comorbid generalized anxiety disorder and MDD (12). However, a recent review highlights that the current setup of dual active treatments combining tDCS with psychological interventions may not achieve increased efficacy in MDD as compared to stand-alone interventions, possibly due to a lack of a full-factorial design (i.e., control psychological intervention), small sample sizes, high variability in study characteristics (e.g., number of sessions, type of psychological intervention), and individual patient characteristics (e.g., brain state at time of stimulation) (10). Additionally, recent studies failed to find the superior efficacy of concurrent tDCS and CBT (13) or concurrent tDCS and other psychosocial interventions (14) when compared with stand-alone treatments, warranting further evidence to the field.

Here, we build on current literature and present the effects of Flow™ combined with psychotherapy and medication on depression and anxiety symptoms, in seven patients presenting MDD, illness anxiety disorder, obsessive-compulsive disorder (OCD), and anxiety disorders. Flow™ offers the possibility of a dual active treatment (tDCS and an app offering evidence-based behavioral therapy sessions), while being a patient-friendly device with no physical restraints. It further provides psychoeducational materials and enables long-distance supervision, through its web-based clinicians dashboard which differentiates Flow™ from other home-based solutions.

## Methods

### Participants

This case series reports retrospective data from seven patients attending a private healthcare clinic for treatment of depressive symptoms, with and without comorbid anxiety or obsessive-compulsive symptoms, between August 2020 and March 2022. Patients provided written informed consent for participation in the intervention protocol and for their individual clinical information to be used.

Patients were diagnosed with MDD and/or other comorbidities by a psychiatrist and/or trained licensed psychologist at baseline and reassessed at week 6 and at the end of treatment following a semi-structured interview based on the Diagnostic and Statistical Manual of Mental Disorders (DSM-V) criteria (15). The self-report version of the Montgomery-Åsberg Depression Rating Scale (MADRS) (16) was further applied as the primary outcome to assess clinical severity across treatment.

The Flow program was introduced to patients who presented mild to moderate depressive symptoms, were resistant to initiate or augment medication, or who showed a preference for non-pharmacological treatments. Following treatment admission, patients started Flow sessions (cf. Supplementary Figure 1) and completed the following questionnaires to assess clinical status and improvement: the self-reported Montgomery-Åsberg Depression Rating Scale (MADRS-S) (17), the Beck Depression Inventory-II (BDI-II) (18, 19), and the State-Trait Anxiety Inventory (STAI) (20, 21). tDCS acceptability was assessed using the ACCEPT-tDCS (22).

Questionnaires were administered at baseline and at the end of weeks 3 and 6 of treatment. MADRS-S was requested at the 1-month follow-up. Patients reported side effects weekly using the Portuguese translation of the Thair et al. questionnaire (23). Side-effect management strategies are reported in section “Adverse Effects Results” of supplementary material. Clinical progress monitoring was performed in-person and remotely using Zoom [Zoom Video Communications, Inc., 2020 (Computer software)], according to individual preference. At the end of week 6, patients were re-assessed and the treatment proceeded according to the patient's choice and clinical recommendation (i.e., to continue in psychotherapy and/or pharmacology as stand-alone treatments when the patient was responding positively to treatment as per self-reports and clinical interview, to start maintenance treatment [when symptoms' remission was achieved (MADRS-S ≤ 12)], or to repeat the Flow program (when clinical response was ongoing but symptoms remission not achieved). The Flow Program schedule can be found in Table 1.

**Table 1:** Flow program treatment schedule.

**Timepoint**	**Screening session**	**Day 1 of week 1**	**Day 1 of week 2**	**Day 1 of week 3**	**Day 1 of week 4**	**Day 1 of week 5**	**Day 5 of week 6**	**Follow-up (1 month)**
Clinical assessment	X							X
Eligibility screening (and monitoring)	X	X	X	X	X	X	X	
Informed consent	X							
MADRS interview	X						X	
ACCEPT-tDCS		X		X			X	
STAI-Y2		X		X			X	
BDI-II		X		X			X	
MADRS-S*		X	X	X	X	X	X	X
Adverse effects questionnaire		X	X	X	X	X	X	
Patient feedback		X	X	X	X	X	X	X

MADRS, Montgomery-Åsberg depression rating scale; ACCEPT-tDCS, transcranial direct current stimulation acceptability in the treatment of anxiety disorders questionnaire; STAI-Y2, subscale trait-anxiety of the state-trait anxiety inventory (form Y); BDI-II, beck depression inventory-II; MADRS-S, self-report version of the MADRS; *Completed using the Flow depression app.

Clinically significant change was calculated based on percentage change and the Reliable Change Index (RCI). RCI (24) was assessed using the formula (Xpost–Xpre)/√2 (SD^*^√1–α)^2^, where Xpost is the result post-intervention, Xpre the result at baseline and SD the standard deviation and α the reliability from the corresponding psychometric publications. We adopted the indexes and confidence intervals (CI) by Wise (25) as indicative of clinically significant change: RCI ≥ |1.96|, 95% CI; RCI ≥ |1.64|, 90% CI; RCI ≥ |1.28|, 80% CI.

Patients included four women and three men (26–51 years), of which two were diagnosed with comorbid MDD and anxiety disorder, one with OCD, one with anxiety disorder, two with dysthymia and one with illness anxiety disorder. All patients presented depressive symptomatology at intake. Four patients started cognitive-behavioral therapy (CBT) prior to Flow and maintained concomitantly. Five patients were medication-free and two were on medication at the start of the program. The latter were in stable dose for at least 4 weeks prior to treatment (cf. Supplementary Table 1). Two patients initiated CBT at the same time as Flow.

#### Case 1

Patient 1 was a 41-year-old married woman, with a high education level and stable employment. She presented a history of recurrent major depressive episodes, concomitant to an unspecified anxiety disorder. During her second pregnancy, patient 1 developed moderate MDD (peripartum onset). At intake (6 years after her second pregnancy), she exhibited depressed mood, sadness, irritability, decreased sleep and appetite, and anxiety symptoms (increased physiological activity). No suicidal ideation or suicide attempts were reported. The patient had no history of drug or alcohol abuse and no family history of mental illness. She had sought professional help before for the presenting symptoms and had previously completed one psychotherapy course. Prior to treatment, the patient was medication-free. The patient completed Protocol 1 (18 tDCS sessions).

#### Case 2

Patient 2 was a 22-year-old unmarried young man. At intake, he was a university student and a professional football athlete. He reported having alopecia for several years and resolved Guillain-Barre syndrome in the past months. He had a history of major depressive episodes, initiating in his childhood. Presenting complaints included persistent depressive symptoms, comorbid with anxiety disorders [specific phobia (heights) and agoraphobia], with a significant impact on his academic and athletic performance. The patient had no history of drug or alcohol abuse and no previous psychiatric admissions but reports a suspected family history of MDD (father). This was the second time the patient sought professional help for the presenting symptoms which were addressed with psychotherapy and pharmacotherapy (sertraline 50 mg). This time the patient's treatment of choice was FLOW. Patient 2 initiated Flow at the same time as psychotherapy and completed Protocol 1 (17 tDCS sessions).

#### Case 3

Patient 3 was a 31-year-old unmarried man with stable employment and a high education level. He presented to the clinic with prior long-term cannabis use associated with withdrawal syndrome with mild depressive symptoms and social anxiety disorder (performance only). No substance use in the present and no psychiatric family history or prior psychiatric events were reported. Symptoms onset occurred at the start of the COVID-19 pandemic. The patient reported no prior attempts of psychotherapy or pharmacotherapy. Patient 3 initiated Flow simultaneously to CBT, having completed 25 sessions (Protocol 2, with maintenance phase).

#### Case 4

Patient 4 was a 37-year-old single woman with a high education level and unstable employment. She presented comorbid depressive and anxious symptoms at intake (depressed mood, irritability, feelings of worthlessness and guilt, reduced attention, muscular tension), emerging during adolescence. She was previously diagnosed with persistent depressive disorder (dysthymia) and medicated with Vortioxetine, without improvement. Afterward, she initiated Bupropion (150 mg), Quetiapine (25 mg), and Bromazepam (1.5 mg in SOS). The patient reported no history of drug or alcohol consumption. Also, she reported no prior psychotherapeutic treatments. Psychiatric family history included an aunt diagnosed with MDD and her grandmother with suspected MDD. Patient 4 was diagnosed with dyslexia early at school age but never benefited from any formal support. The patient initiated Flow concomitantly to CBT (24 tDCS sessions; Protocol 2, with maintenance phase).

#### Case 5

Patient 5 was a 27-year-old unmarried woman. At intake, she was a university student with simultaneous stable employment. She presented depressive symptoms (diminished ability to think and indecisiveness, lack of energy) associated with episodes of binge eating and was diagnosed with dysthymia. No previous resolution attempts were reported. Although no family history of mental illness was observed, the patient highlighted psychosocial impairments, namely family conflict and difficulty in establishing boundaries. Patient 5 completed two consecutive acute cycles of Flow treatment simultaneously with psychotherapy (Protocol 2, 39 tDCS sessions; reasons detailed below).

#### Case 6

Patient 6 was a 27-year-old unmarried woman, in her last doctoral years. She presented an illness anxiety disorder, emerging in early childhood (4 years old) and currently comorbid with depressive symptomatology (loss of appetite, loss of interest). Symptoms were associated with avoidance behaviors related to fear of contamination. Although not diagnosed, a family history of illness anxiety disorder was suspected (father). This was the first time the patient sought professional help. No relevant medical background was reported, except a weakened immune system with recurrent candidiasis. Patient 6 completed two independent cycles of Flow (Protocol 1, 18 tDCS sessions each), at two distinctive episodes 3 months apart, simultaneously with CBT. During the second cycle, patient 6 also initiated pharmacotherapy.

#### Case 7

Patient 7 was a 51-year-old married man with an intermediate level of education and stable employment, diagnosed with OCD. At intake, he was in psychotherapy and medicated with Sertraline (100 mg), Clomipramine (75 and 25 mg), and Clonazepam (0.5 mg), in another clinic. The patient was referred for Flow as a complementary treatment to manage severe depressive symptoms causing significant distress. Patient 7 completed 18 tDCS sessions (Protocol 1).

### Intervention

Flow (Flow Neuroscience AB, Sweden) combines self-administrated tDCS with a smartphone app (Flow Depression) for behavioral therapy, aiming to activate neural networks and implement healthy habits and contribute to the reduction of depressive symptoms. Flow app is combined with a certified tDCS medical device approved for home-use MDD treatment in adult patients (>18 years old) in the United Kingdom and the European Union. The one-size-fits-all wireless and portable tDCS headset targets the prefrontal cortex (the anode electrode over the left and the cathode electrode over the right dorsolateral prefrontal cortex; cf., Supplementary Figures 2, 3), as evidenced by electric field modeling (26). The device uses a current intensity of 2 mA, administered through two spheric electrodes of 22.9 cm^2^ size (current density = 0.09 mA/cm^2^) for 30 min.

After clinical studies evidence showing the beneficial effect of 15 consecutive sessions in depression (27, 28), Flow updated the number of sessions during the acute treatment, transitioning from Protocol 1 (acute treatment phase for 2 weeks) to Protocol 2 (acute treatment phase for 3 weeks). The protocols consisted of an acute phase of daily sessions, five sessions per week during the first 2 weeks (Protocol 1) or the first 3 weeks (Protocol 2), followed by a maintenance phase of twice-weekly sessions for 4 or 3 weeks, respectively (18 or 21 sessions in total, for a total of 6 weeks). According to the manufacturers, the maintenance phase can be extended up to week 10.

Patients were introduced to Flow and trained by a clinical psychologist certified in tDCS. Weekly appointments with the psychologist allowed to monitor clinical progression, discuss treatment adherence, answer patients' questions, and collect self-reported adverse effects.

The app offers automated virtually guided behavioral therapy sessions developed by licensed clinical psychologists. The different courses focus on behavioral activation, sleep hygiene, mindfulness-based meditation, physical exercise, and nutrition. Sessions can be completed during the 30 min stimulation, and are not mandatory. Upon patient's approval, a dashboard for clinicians is currently available to monitor clinical progression and adherence.

To initiate Flow, eligibility criteria were verified across time. Exclusion criteria were followed according to recommendations in the field (6) (cf. Supplementary Table 1) and assessed using the Exclusion Criteria Questionnaire for tDCS (23).

## Clinical findings/results

### Depression and anxiety symptoms

**Figure 1:**
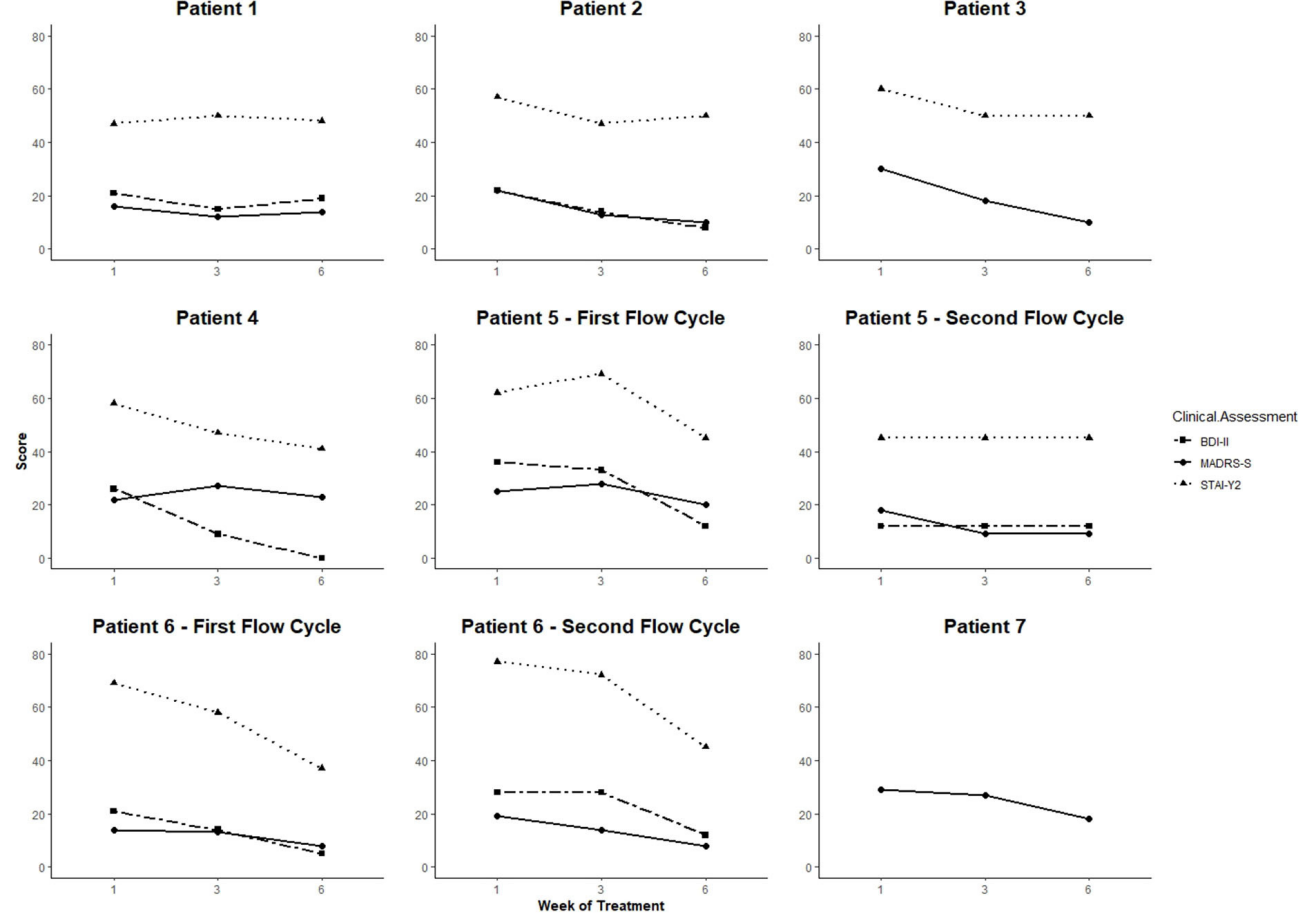
MADRS-S, BDI-II, and STAI-Y2 results by patient across the Flow treatment. X-axis shows measuring time points, y-axis shows scores. MADRS-S results were not available for patient 7. MADRS interview performed by the clinician is depicted as a proxy value.

MADRS-S, BDI-II, and STAI-Y2 scores from baseline to week 6 of treatment are shown in Figure 1. Percentage change scores and Reliable Change Index (RCI) are reported in Table 2. At the end of week 76, five patients showed clinical improvement for depressive symptoms using MADRS-S (percentage change: 37.9–66.7%; RCI: −1.45, 80% CI; RCI: −2.17 to −4.82, 95% CI) and four using BDI-II (percentage change: 57.1–100%; RCI: −3.61 to −6.70, 95% CI). Five patients presented significant improvement in anxiety symptoms (STAI-Y2 percentage change: 12.3–46.4%; RCI: −1.79, 90% CI; RCI: −2.55 to −8.64, 95% CI). One patient (Patient 1) did not respond to treatment. Patients that presented significant clinical improvements combined Flow with CBT and/or psychopharmaceuticals.

**Table 2:** Clinical findings before and after 6 weeks of treatment.

**Patient**	**Diagnosis**	**MADRS-S**				**STAI-Y2**				**BDI-II**				**Flow protocol**	**Total tDCS sessions**	**Dual active treatment**
		**Baseline**	**Week 6**	**Percentage change**	**RCI**	**Baseline**	**Week 6**	**Percentage change**	**RCI**	**Baseline**	**Week 6**	**Percentage change**	**RCI**			
**1st FLOW cycle**															
Patient 1	MDD and unspecified AD	16	14	−12.5%	−0.48	47	48	2.1%	0.27	21	19	−9.5%	−0.52	1	18	Flow stand-alone
Patient 2	MDD and Agoraphobia + Specific Phobia	22	10	−54.5%	−2.89***	57	50	−12.3%	−1.79**	22	8	−63.6%	−3.61***	1	17	Flow and CBT
Patient 3	Social AD (performance)	30	10	−66.7%	−4.82***	60	50	−16.7%	−2.55***	27	N/A	N/A	N/A	2	25	Flow and CBT
Patient 4	Dysthymia	22	23	4.5%	0.24	58	41	−29.3%	−4.59***	26	0	−100%	−6.70***	2	24	Flow, CBT and antidepressant/ benzodiazepine (bupropion 150 mg; quetiapine 25 mg; bromazepam 1.5 mg in SOS)
Patient 5	Dysthymia	25	20	−20%	−1.20	62	45	−27.4%	−4.59***	36	12	−66.7%	−6.18***	2	23	Flow and CBT
Patient 6	Illness AD	14	8	−42.9%	−1.45*	69	37	−46.4%	−8.64***	21	5	−76.2%	−4.12***	1	18	Flow and CBT
Patient 7 ^a^	OCD	29	18	−37.9%	−2.65***	40	N/A	N/A	N/A	33	N/A	N/A	N/A	1	18	Flow, CBT and antidepressant/ benzodiazepine (sertraline 100 mg, clomipramine 75 mg and 25 mg; clonazepam 0,5 mg)
**2nd FLOW cycle**															
Patient 5	Dysthymia	18	9	−50%	−2.17***	45	45	0%	0	12	12	0%	0	2	16	Flow and CBT
Patient 6	Illness AD	19	8	−57.9%	−2.65***	77	45	−41.6%	−8.64***	28	12	−57.1%	−4.12***	1	18	Flow, CBT and antidepressant at week 4 (Escitalopram 10 mg)

MADRS-S, self-report version of the Montgomery-Åsberg depression rating scale; STAI-Y2, subscale trait-anxiety of the state-trait anxiety inventory (form Y); BDI-II, beck depression inventory-II; Baseline, Pre-Treatment; Percentage Change, ((Week 6–Baseline)/Baseline)^*^100; RCI, reliable change index (improvement from Baseline to week 6; difference between week 6 and Baseline divided by the standard error of the difference for the test); MDD, major depressive disorder; AD, anxiety disorder; CBT, cognitive-behavioral therapy; N/A, not available; OCD, obsessive-compulsive disorder.

^a^ MADRS-S results were not available for patient 7 (missing value). Accordingly, we used the MADRS interview administered by the clinician as a proxy value.

RCI significance levels: ***RCI ≥ |1.96|, 95% CI; **RCI ≥ |1.64|, 90% CI; *RCI ≥ |1.28|, 80% CI.

According to clinical decisions and patients' preferences, patients 3 and 4 were recommended for eight additional tDCS sessions after the maintenance phase (until week 10) to consolidate clinical response. However, both completed only four sessions across 4 weeks. During the maintenance phase, we registered a significant improvement between weeks 6–10 in anxiety symptoms for patient 3 (STAI-Y2 percentage change: −30%) and depression symptoms for patient 4 (MADRS-S percentage change: −60.87%; cf. Supplementary Table 2).

Patients 5 and 6 initiated two cycles. Patient 6 started the second cycle 3 months after the first treatment due to the re-emergence of depression symptoms. This second course had a significant impact on depression and anxiety symptoms with decreased percentage changes between 41.6 and 57.9% (cf. Table 2). Patient 5 initiated the second cycle after 5 weeks of reduced adhesion to treatment. The second course was significantly associated with symptom improvement at 6 weeks as assessed by MADRS-S (percentage change: −50%; RCI: −2.17, 95% CI), but not as assessed by BDI-II and STAI-Y2 (cf. Table 2).

Across patients, improvement of depression and anxiety symptoms was maintained at 1-month follow-up (cf. Supplementary Table 3). Having completed Flow treatments, six patients (except patient 1) maintained weekly to once-a-month psychotherapy. Two patients initiated Escitalopram (10 mg): patient 6 during the second cycle as her anxiety symptoms became the primary concern, associated with ritual behaviors, and patient 1 after the lack of response to the Flow program. Patients 4 and 7 maintained their antidepressants and benzodiazepines.

### Intervention adherence and compliance

Patients' adherence and acceptability were overall high (76.2–100%; cf. ACCEPT-tDCS scores in Supplementary Table 2). Patients 3 and 4 reported personal challenges that negatively influenced the treatment process which led to 50% missed tDCS sessions during the maintenance phase. Considering the minimal improvement presented by these patients, missed sessions were not compensated. Patient 5 did not comply with the prescribed treatment and dropped out after the first 3 weeks of Flow. Data regarding adherence to the app was available for three patients through the clinician's dashboard. Only one patient completed the courses proposed by the app consistently (cf. Supplementary Table 2). Finally, follow-up assessments at 1 month for three patients are not available.

tDCS was well-tolerated, without severe side effects (cf. Supplementary Table 4). Our observed side effects are in line with the tDCS literature, and no unexpected events were reported. The most common adverse effects were scalp irritation, tingling, itching, and burning sensation. Patient 4 reported high levels of back and neck pain, attributed to the seated position while completing tDCS sessions and to muscles' tension (an anxiety symptom reported by this patient) and not a direct effect of stimulation. No patient interrupted the tDCS treatment due to the side effects.

## Discussion

This case series explored the effect of the Flow Program combined with psychotherapy and/or pharmacotherapy in seven patients affected by depressive and anxious disorders. Overall, we found mood and anxiety improvement after treatment, except for one MDD patient who was not undergoing simultaneous psychotherapy or pharmacotherapy. tDCS efficacy is promising in dysphoric and psychomotor retardation symptoms of depression but not in vegetative/somatic symptoms (29). Patient 1's non-response to tDCS may be associated with her somatic depression related to dysfunction of the autonomic nervous system, and not the prefrontal cortex (30).

Our findings are in line with previous case series (11) and may be explained by synergistic effects on neuroplasticity of combining tDCS and individually tailored psychotherapy (10). Both tDCS and psychological interventions have the potential to restore basic and higher-order psychological mechanisms (31). Specifically, tDCS can be used to facilitate learning of cognitive control and emotional and behavioral regulation, targeting adaptive processes and restoring brain functioning in the prefrontal cortex (10, 31, 32). Consequently, patients' benefit from psychotherapy increases, as it requires higher-order cognitive processes frequently impaired in depressed and anxious patients (31). In our case series, patient 2 was not benefiting from CBT prior to Flow. After 6 weeks of Flow, he manifested significant improvement in both depressive and anxious symptomatology, which was maintained at the 1-month follow-up.

Although the results of dual active treatments of tDCS with antidepressants are conflicting [e.g., lower depression scores and higher response rates (33) vs reduced antidepressant effect of tDCS when combined with benzodiazepines (34, 35)], warranting new clinical studies to unveil treatment parameters, the potential benefit of tDCS combined with antidepressants was preliminarily observed in our patients 4 and 7, with a reduction of depression and anxiety scores. Moreover, our findings seem to contrast with the literature reporting the lack of effect of tDCS combined with psychotherapy (33) which might be due to differences in stimulation parameters. The observed improvements during the maintenance phase are also in accordance with dosage-dependent tDCS effects and the need for short intervals in the post-acute treatment of depression (36–38), suggesting that longer treatment courses may lead to optimal results (5). Finally, our study highlights home-based tDCS safety profile.

Dual active treatments seem to improve in parallel depressive symptoms and trait-anxiety (although to a lesser extent) across patients. This is supported by the neural commonalities between depression and anxiety described by Maggioni et al. (39) that suggested that clinical similarities between MDD and anxiety could be attributed to shared alterations in prefrontal regions, associated with emotional processing and regulation. Consequently, targeting the prefrontal cortex with tDCS concurrently with other treatments may result in greater cognitive and emotional regulation and subsequent reduced depressive and anxiety symptoms (12).

Brain-derived neurotrophic factor (BDNF) is a key regulator of neuronal growth and survival, contributing to neural function and plasticity (40). It has frequently been proposed that BDNF lower expression has a role in the pathophysiology of MDD (41). Although with inconsistent results, it has emerged as an important mechanism associated with antidepressant clinical response (41). Also, longer-lasting tDCS-elicited changes in synaptic plasticity may involve BDNF-mediated mechanisms (42). Studies on the relationship between tDCS effects and elevated BDNF levels after treatment in depressed patients have shown conflicting results thus far with BDNF plasma levels not increasing following tDCS (43). This suggests that whereas BDNF levels might not be impacted by tDCS treatments, pre-treatment BDNF levels can be a predictor of treatment response. In fact, a similar effect was seen with psychotherapy by the study from Bruijniks et al. (44) which observed that higher levels of BDNF at baseline were related to lower post-treatment depression although only in patients with high working memory.

For patients 4 and 5, improvement in depression scores for MADRS-S and BDI-II were incongruent. Although BDI-II and MADRS-S are self-assessment depression screening measures, with sufficient agreement between them, they are also different in several aspects. Compared with the Beck Depression Inventory (BDI-I), MADRS-S has been found to be less influenced by maladaptive personality traits and more focused on core depressive symptoms and states. Consequently, MADRS-S has been recommended to discriminate state depressiveness in mild depression and coexisting personality traits (45). Additionally, the two measures report distinctive time windows (the past 3 days vs the past 2 weeks) and use distinctive response systems (fixed sentences vs fixed sentences interleaved with open scores) leading to different reports of the phenomenological processes.

An increased interest in home-based tDCS solutions has been growing as it removes the disadvantages of in-person visits (46, 47). Our results show not only its promising early antidepressant effects but also the high rates of treatment adherence, potentiated by comprehensive training and remote supervision (37). Such findings further drive our recommendation of tDCS as an alternative treatment for patients who cannot or do not wish to take medication (e.g., pregnant women) (30), broadening treatment decisions while increasing patients' self-management of their mental health.

To support patients in the management of their own treatment and adverse effects, a thorough informational stage concerning what is expected during treatment is needed. This stage offers patients the perception of control and adds to their perception of self-efficacy managing their mental health. Additionally, a close access by the patient to the health professional is critical. In the current case series, we describe a set of case studies where patients were instructed to reach out to their health professional by WhatsApp (text or phone call) at any time during the first week in case of adverse effects or to answer any question concerning the treatment. From there, patients were able to discuss side effects and worries during the weekly sessions. Of interest, our experience shows that although available, most patients do not request daily support to manage treatment delivery nor side effects in the first week. However, from their feedback, patients feel well-supported with this option as well as welcome open discussions about their experience during the weekly sessions.

This case series offers a report of real-context dual active treatments that include home-based tDCS. This study has several limitations worth considering. It lacks strategies to control bias and follow-up assessments were not available for all patients, compromising a better overview of the long-term impact of the treatment. Additionally, most patients have a high-education level and possibly a high cognitive reserve and learning capacity, which might be positive bias to the effects of the dual active treatment. Patients also presented heterogeneous symptoms and treatment protocols (i.e., variable concomitant adjunct pharmacotherapy and psychotherapy). Clinical outcomes were based on self-reported measures, which in a clinical sample with cognitive deficit/biases warrants consideration. Finally, difference in the mode of tDCS administration may be an additional source of variability. Further randomized trials using home-based tDCS are needed to establish its efficacy as a stand-alone or part of dual active treatments.

## Patient perspective

Patients' perspectives collected through an anonymous online survey showed that both the Flow Depression App, tDCS sessions, and weekly appointments with the clinician assisted in symptom reduction. The most positive aspects of treatment were the almost immediate effects felt and maintained across time; the equipment portability and ease of use; and the app providing tools for everyday life challenges. One patient highlighted that the combination of different treatment strategies has led to an optimized result. Tingling sensation and discomfort during stimulation were the only negative experiences reported in this survey. However, only 3 of the 7 patients replied to the survey, which may reflect a positive bias and absence of negative feedback in the patient's perspective. Considering the reduced/absent therapeutic response in some of the cases and the adverse side effects experienced, we cannot discount the existence of unreported negative experiences in the case series. For example, in patient 1, the acceptability of tDCS reduced from week 1 to week 6, while for others there is a positive slope on treatment acceptability across the treatment protocol (cf. Supplementary Table 2).

## Data availability statement

The original contributions presented in the study are included in the article/Supplementary material, further inquiries can be directed to the corresponding author.

## Ethics statement

Ethical review and approval was not required for the study on human participants in accordance with the local legislation and institutional requirements. The patients/participants provided their written informed consent to participate in this study. Written informed consent was obtained from the individual(s) for the publication of any potentially identifiable images or data included in this article.

## Author contributions

MS, RG, VM, and AG-Á: conceptualization, formal analysis, investigation, and writing-original draft preparation. MS, RG, and AG-Á: methodology. MS and AG-Á: validation. RG, VM, and AG-Á: writing-review and editing. MS: visualization. AG-Á: supervision. All authors have read and agreed to the published version of the manuscript.

## Funding

RG and MS are supported by a Ph.D. Grant (SFRH/BD/5099/2020; 2021.07006.BD, respectively) and sponsored by the Portuguese Foundation for Science and Technology. AG-Á is supported by the Portuguese Foundation for Science and Technology [Individual Call to Scientific Employment Stimulus−3rd Edition 2019-−2020.02059.CEECIND]. The Center for Research in Neuropsychology and Cognitive and Behavioral Intervention (CINEICC) of the Faculty of Psychology and Educational Sciences of the University of Coimbra is supported by the Portuguese Foundation for Science and Technology and the Portuguese Ministry of Education and Science through national funds and co-financed by FEDER through COMPETE2020 under the PT2020 Partnership Agreement [UID/PSI/01662/2013]. Flow Neuroscience supported the payment of the Open-Access fee.

## Conflict of interest

Author(s) MS and VM were employed by Neuroncircuit. AG-A had non-financial support from Soterix and commercial interests with Flow Neuroscience tDCS equipment during the study.

The remaining authors declare that the research was conducted in the absence of any commercial or financial relationships that could be construed as a potential conflict of interest.

## Publisher's note

All claims expressed in this article are solely those of the authors and do not necessarily represent those of their affiliated organizations, or those of the publisher, the editors and the reviewers. Any product that may be evaluated in this article, or claim that may be made by its manufacturer, is not guaranteed or endorsed by the publisher.
