# Supplementary material for: Home-based transcranial direct current stimulation in dual active treatments for symptoms of depression and anxiety: A case series
Source: Front Psychiatry. 2022 Oct 6;13:947435. doi: 10.3389/fpsyt.2022.947435 (PMC9583668; doi:10.3389/fpsyt.2022.947435)
Supplement: Supplementary file 1 [file Data_Sheet_1.docx]

Supplementary Material

Home-based transcranial direct current stimulation in dual active treatments for symptoms of depression and anxiety: A case series

Mónica Sobral^1,2^, Raquel Guiomar^1*^, Vera Martins^2,3^, Ana Ganho-Ávila^1*^

^1^Faculty of Psychology and Educational Sciences, Center for Research in Neuropsychology and Cognitive Behavioral Intervention, University of Coimbra, Coimbra, Portugal

^2^Neuroncircuit - e.Stim Clínica de Saúde Mental, Coimbra, Portugal

^3^Coimbra Hospital and University Centre, Coimbra, Portugal

*** Correspondence:**Ana Ganho-Ávila
[ganhoavila@fpce.uc.pt](mailto:ganhoavila@fpce.uc.pt)

**This document includes:**

1. Supplementary Figures

Figure S1. Flow-Chart of the Flow Program

Figure S2. Flow tDCS device

Figure S3. Flow Depression App

1. Supplementary Tables

Table S1. Exclusion Criteria

Table S2. Flow program results

Table S3. Improvement between pre- and post- dual active treatments with Flow

Table S4. Adverse effects results

# Supplementary Figures

## Flow-Chart of the Flow Program

##
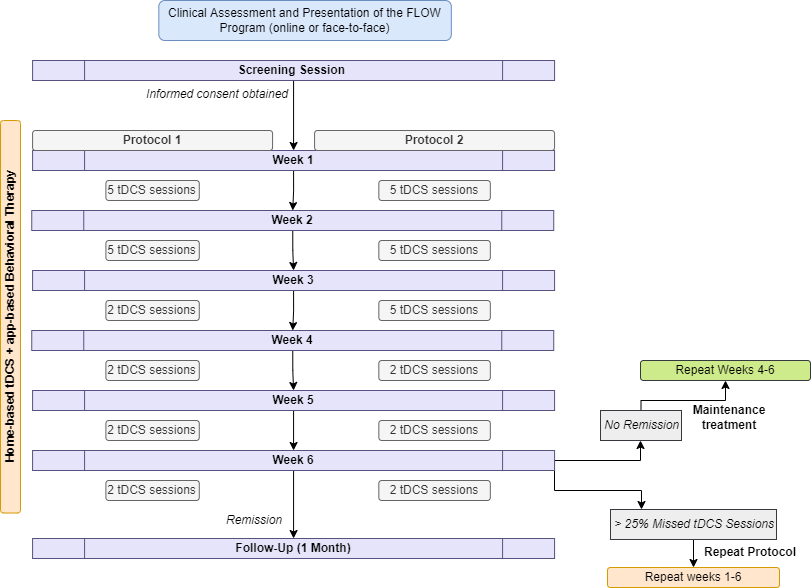


## Figure S1. Flow-Chart showing the treatment schedule of the FLOW program.

## Flow tDCS and app devices


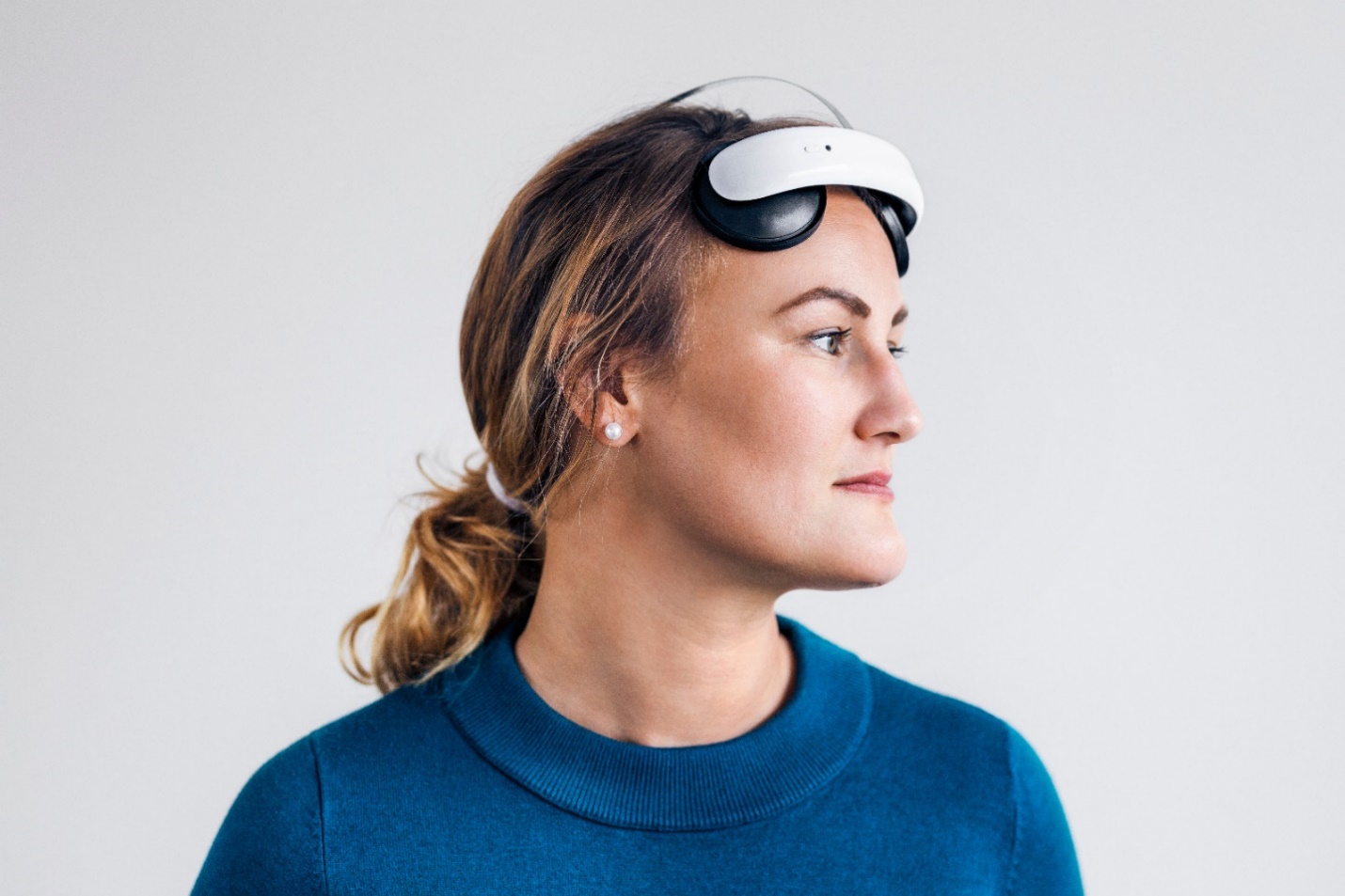
**Figure S2.** Flow tDCS device, with the anode electrode over the left and the cathode electrode over the right dorsolateral prefrontal cortex.


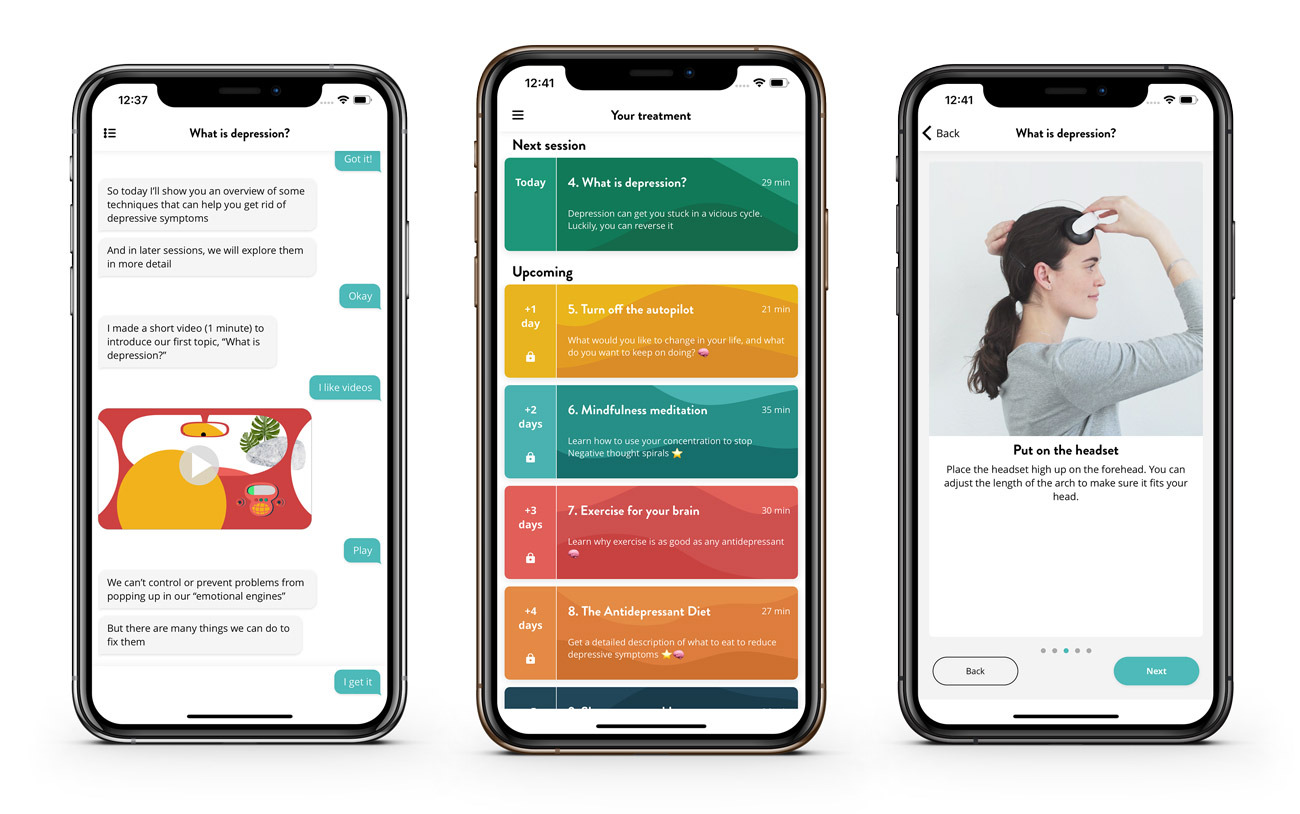


**Figure S3.** The Flow Depression App. A virtual therapist assists patients with their treatment plan, schedules upcoming sessions, assigns homework, and tracks depression symptoms using MADRS-S. The program includes seven courses, with over 50 behavioral therapy sessions, designed to implement antidepressant habits and strategies. The courses focus on physical exercise (e.g., the most beneficial type of exercise and appropriate “dose”); meditation (e.g., practical exercises); sleep (e.g., sleep hygiene and its relationship with depression); nutrition (e.g., healthy eating habits to reduce inflammation in the brain).

# Supplementary Tables

## Exclusion Criteria

**Table S1.** Detailed eligibility criteria

| **Exclusion Criteria** | |
| --- | --- |
| Age lower than 18 years old | |
| Direct relatives (parents or siblings) diagnosed with epilepsy | |
| Head injury resulting in loss of consciousness that required further investigation (e.g., brain scan) | |
| Diagnosis of a psychological disorder other than MDD, illness anxiety disorder, anxiety disorders or OCD (e.g., bipolar disorder) | |
| Diagnosis of neurological condition (e.g., epilepsy, seizure, migraines) incompatible with tDCS treatment, according to the clinician assessment | |
| Diagnosis of cardiac conditions | |
| Metal implants in the head (including mouth), such as shrapnel or surgical clips | |
| Device implanted in head or pacemaker | |
| Skin condition on the scalp (e.g., psoriasis) or head injuries/scars | |
| Prior adverse reactions to tDCS or other brain stimulation techniques | |
| Self-medicating, such as recreational drugs | |
| Taking specific prescribed medications | |
| **Medication included** | |
| Anxiolytic benzodiazepines | Alprazolam, Bromazepam, Lorazepam, Medazepam, Oxazepam |
|  | Cetazolam, Clobazam, Cloxazolam, Diazepam, Halazepam, Mexazolam, Prazepam |
|  | Brotizolam, Midazolam, Triazolam, Zolpidem |
| Hypnotic benzodiazepines | Flurazepam, Quazepam |
| Tricyclic antidepressants | Clomipramine, Amitriptyline, Desipramine, Imipramine, Nortriptyline |
| Selective serotonin reuptake inhibitors (SSRIs) | Fluoxetine, Paroxetine, Sertraline, Citalopram |
| Monoamine oxidase inhibitors (MAOIs) | Phenelzine (Nardil), Isocarboxazid (Marplan), Tranylcypromine (Parnate) |
| Atypical antidepressants | Bupropion, Trazodone, Amoxapine, Maprotiline |
| **Medication not included (exceptions allowed under clinicians’ decision)** | |
| Antiepileptic/anticonvulsant benzodiazepines | Clonazepam |
| Anticonvulsants and antiepileptics | Carbamazepine, Primidone, Valproic acid, Phenytoin, Oxcarbazepine |
| Antiparkinson | Levodopa |
| Mood stabilizers | Lithium, Carbamazepine, Lamotrigine, Divalproex, Topiramate |
| Antipsychotics | Risperidone, Clozapine, Olanzapine, Quetiapine, Chlorpromazine, Haloperidol |

## Flow program results

**Table S2.** Detailed results of the Flow program

| **Patient** | **tDCS sessions** | **Treatment Week** | **MADRS-S** | **BDI-II** | **Classification** | **STAI-Y2** | **ACCEPT-tDCS** | **Flow Depression App** |
| --- | --- | --- | --- | --- | --- | --- | --- | --- |
| Patient 1 | 18 (% of completed sessions: 100%) | Week 1 | 16 | 21 | Moderate depression | 47 | 83 | NA |
|  |  | Week 2 | 12 | - | - | - | - |  |
|  |  | Week 3 | 12 | 15 | Mild depression | 50 | 79 |  |
|  |  | Week 4 | 12 | - | - | - | - |  |
|  |  | Week 5 | 10 | - | - | - | - |  |
|  |  | Week 6 | 14 | 19 | Mild depression | 48 | 69 |  |
|  |  | Follow-up (1 month) | NA | - | - | - | - |  |
| Patient 2 | 17 (% of completed sessions: 94.4%) | Week 1 | 22 | 22 | Moderate depression | 57 | 79 | NA |
|  |  | Week 2 | 14 | - | - | - | - |  |
|  |  | Week 3 | 13 | 14 | Mild depression | 47 | 78 |  |
|  |  | Week 4 | 10 | - | - | - | - |  |
|  |  | Week 5 | 10 | - | - | - | - |  |
|  |  | Week 6 | 10 | 8 | Minimal | 50 | NA |  |
|  |  | Follow-up (1 month) | 11 | - | - | - | - |  |
| Patient 3 | 25 (% of completed sessions: 86.2%) | Week 1 | 30 | 27 | Moderate depression | 60 | 80 | Completed courses: 1  Completed sessions: 18  Homeworks done: 2 |
|  |  | Week 2 | 30 | - | - | - | - |  |
|  |  | Week 3 | 18 | NA | NA | 50 | 81 |  |
|  |  | Week 4 | 13 | - | - | - | - |  |
|  |  | Week 5 | 12 | - | - | - | - |  |
|  |  | Week 6 | 10 | NA | NA | 50 | 81 |  |
|  |  | Week 10 | 8 | 3 | Minimal | 35 | 86 |  |
|  |  | Follow-up (1 month) | NA | - | - | - | - |  |
| Patient 4 | 24 (% of completed sessions: 82.8%) | Week 1 | 22 | 26 | Moderate depression | 58 | 75 | Completed courses: 6  Completed sessions: 81  Homeworks done: 10 |
|  |  | Week 2 | 13 | - | - | - | - |  |
|  |  | Week 3 | 27 | 9 | Minimal | 47 | 74 |  |
|  |  | Week 4 | 18 | - | - | - | - |  |
|  |  | Week 5 | 12 | - | - | - | - |  |
|  |  | Week 6 | 23 | 0 | Minimal | 41 | 62 |  |
|  |  | Week 10 | 9 | 0 | Minimal | 41 | 72 |  |
|  |  | Follow-up (1 month) | 12 | - | - | - | - |  |
| Patient 5 – Cycle 1 | 23 (% of completed sessions: 82.1%) | Week 1 | 25 | 36 | Severe depression | 62 | 80 | Completed courses: 1  Completed sessions: 20 |
|  |  | Week 2 | 27 | - | - | - | - |  |
|  |  | Week 3 | 28 | 33 | Severe depression | 69 | NA |  |
|  |  | Week 4 | 24 | - | - | - | - |  |
|  |  | Week 5 | 25 | - | - | - | - |  |
|  |  | Week 6 | 20 | 12 | Minimal | 45 | NA |  |
| Patient 5 – Cycle 2 | 16 (% of completed sessions: 76.2%) | Week 1 | 18 | 12 | Minimal | 45 | NA |  |
|  |  | Week 2 | 16 | - | - | - | - |  |
|  |  | Week 3 | 9 | 12 | Minimal | 45 | NA |  |
|  |  | Week 4 | 9 | - | - | - | - |  |
|  |  | Week 5 | 9 | - | - | - | - |  |
|  |  | Week 6 | 9 | 12 | Minimal | 45 | NA |  |
|  |  | Follow-up (1 month) | 6 | - | - | - | - |  |
| Patient 6 – Cycle 1 | 18 (% of completed sessions: 100%) | Week 1 | 14 | 21 | Moderate depression | 69 | 86 | NA |
|  |  | Week 2 | NA | - | - | - | - |  |
|  |  | Week 3 | 13 | 14 | Mild depression | 58 | 87 |  |
|  |  | Week 4 | NA | - | - | - | - |  |
|  |  | Week 5 | NA | - | - | - | - |  |
|  |  | Week 6 | 8 | 5 | Minimal | 37 | NA |  |
|  |  | Follow-up (1 month) | 12 | - | - | - | - |  |
| Patient 6 – Cycle 2 | 18 (% of completed sessions: 100%) | Week 1 | 19 | 28 | Moderate depression | 77 | NA | NA |
|  |  | Week 2 | 16 | - | - | - | - |  |
|  |  | Week 3 | 14 | 28 | Moderate depression | 72 | NA |  |
|  |  | Week 4 | 10 | - | - | - | - |  |
|  |  | Week 5 | 14 | - | - | - | - |  |
|  |  | Week 6 | 8 | 12 | Minimal | 45 | NA |  |
|  |  | Follow-up (1 month) | 8 | - | - | - | - |  |
| Patient 7* | 18 (% of completed sessions: 100%) | Week 1 | 29 | 33 | Severe depression | 40 | NA | NA |
|  |  | Week 2 | - | - | - | - | - |  |
|  |  | Week 3 | 27 | NA | NA | 39 | NA |  |
|  |  | Week 4 | - | - | - | - | - |  |
|  |  | Week 5 | - | - | - | - | - |  |
|  |  | Week 6 | 18 | NA | NA | NA | NA |  |
|  |  | Follow-up (1 month) | NA | - | - | - | - |  |

***Note:*** MADRS-S = self-report version of the Montgomery-Åsberg Depression Rating Scale; BDI-II = Beck Depression Inventory-II; Classification = BDI-II cut-off scores: 0 to 13 (minimal), 14 to 19 (mild depression), 20 to 28 (moderate depression), higher than 29 (severe depression); STAI-Y2 = Subscale Trait-Anxiety of the State-Trait Anxiety Inventory (Form Y); ACCEPT-tDCS = Acceptability towards transcranial direct current stimulation self-report questionnaire. Scores for ACCEPT-tDCS were calculated considering the 15-item structure of the questionnaire (total scores from 15 to 90); Flow Depression App = data collected by the clinicians’ dashboard, made available only for patients 3, 4., and 5; NA = not available. *MADRS-S results were not available for patient 7 and MADRS interview by the clinician was used as a proxy value.

## Improvement between pre- and post- dual active treatments with Flow

**Table S3.** Results of Flow program between pre- (baseline) and post-treatment (one-month follow-up)

| **Patient** | **Total tDCS Sessions** | **Dual Active**  **Treatment** | **MADRS-S** | | | |
| --- | --- | --- | --- | --- | --- | --- |
|  |  |  | **Baseline** | **Follow-up** | **Percentage Change** | **RCI** |
| Patient 1 | 18 | Flow stand-alone | 16 | NA | NA | NA |
| Patient 2 | 17 | Flow and CBT | 22 | 11 | -50% | -2.65*** |
| Patient 3 | 25 | Flow and CBT | 30 | NA | NA | NA |
| Patient 4 | 24 | Flow, CBT and antidepressant/benzodiazepine | 22 | 12 | -45.5% | -2.41*** |
| Patient 5 ^a^ | 39 | Flow and CBT | 25 | 6 | -76% | -4.58*** |
| Patient 6 | 18 (Cycle 1) | Flow and CBT | 14 | 12 | -14.3% | -0.48 |
| Patient 6 | 18 (Cycle 2) | Flow, CBT and antidepressant | 19 | 8 | -57.9% | -2.65*** |
| Patient 7 ^b^ | 18 | Flow, CBT and antidepressant/benzodiazepine | 29 | NA | NA | NA |

***Note.*** MADRS-S = self-report version of the Montgomery-Åsberg Depression Rating Scale; Baseline = Pre-Treatment; Follow-up = MADRS-S scores at one month post-treatment; Percentage Change = ((Follow-up-Baseline)/Baseline)*100; RCI = Reliable Change Index (improvement from Baseline to Follow-up; difference between Follow-up and Baseline divided by the standard error of the difference for the test); NA = not available; CBT = Cognitive-Behavioral Therapy;

^a^As patient 5 underwent two consecutive Flow courses, the baseline corresponds to week 1 of the first Flow cycle and the follow-up to the post-treatment after the completion of 39 tDCS sessions.

^b^ MADRS-S results were not available for patient 7 (missing value). Accordingly, we used the MADRS interview administered by the clinician as a proxy value.

RCI significance levels: ***RCI ≥ |1.96|, 95% CI

## Adverse Effects Results

**Management of Adverse Effects.** Our side-effect management strategies consisted in 1) providing psychoeducation regarding possible side effects and the monitoring plan; 2) administration of the weekly standardized questionnaire assessing side effects; 3) if patients presented moderate to significant side effects that disrupt functioning or cause discomfort, the clinician provided suggestions to minimize these during the weekly sessions (e.g., pressing the electrodes for a few seconds to ease tingling). Also, in the first three days of tDCS, daily support was offered to the patient, whenever necessary by phone. Patients were also offered the possibility to discontinue tDCS treatment when facing side effects. Additionally, any report of serious adverse events would result in immediate treatment suspension and the adequate report to the company and local authorities according to the national regulations.

**Table S4.** Adverse effects of Flow tDCS sessions

| **Patient** | **Stimulation Week** | **Migraine** | **Neck pain** | **Back pain*** | **Blurred vision** | **Scalp irritation** | **Tingling sensation** | **Itchiness** | **Accelerated heart rate*** | **Burning sensation** | **Hot flashes*** | **Dizziness*** | **Sudden mood swings** | **Fatigue** | **Nervousness** |
| --- | --- | --- | --- | --- | --- | --- | --- | --- | --- | --- | --- | --- | --- | --- | --- |
| Patient 1 | Week 1 | 1 | 1 | 1 | 1 | 3 | 1 | 1 | 1 | 2 | 1 | 1 | 1 | 2 | 1 |
|  | Week 2 | 6 | 1 | 1 | 1 | 1 | 1 | 7 | 1 | 1 | 1 | 1 | 1 | 6 | 1 |
|  | Week 3 | 1 | 1 | 1 | 1 | 1 | 1 | 6 | 1 | 1 | 1 | 1 | 1 | 1 | 1 |
|  | Week 4 | 1 | 1 | 1 | 1 | 1 | 1 | 1 | 1 | 1 | 1 | 1 | 1 | 1 | 1 |
|  | Week 5 | 1 | 1 | 1 | 1 | 1 | 1 | 1 | 1 | 1 | 1 | 1 | 1 | 1 | 1 |
|  | Week 6 | 6 | 1 | 1 | 1 | 1 | 1 | 1 | 1 | 1 | 1 | 1 | 1 | 1 | 1 |
| Patient 2 | Week 1 | 1 | 1 | 1 | 1 | 1 | 1 | 1 | 1 | 5 | 1 | 1 | 1 | 1 | 1 |
|  | Week 2 | 1 | 1 | 1 | 1 | 1 | 1 | 1 | 1 | 4 | 1 | 5 | 1 | 1 | 1 |
|  | Week 3 | 1 | 1 | 1 | 1 | 3 | 1 | 1 | 1 | 3 | 1 | 1 | 1 | 1 | 1 |
|  | Week 4 | 1 | 1 | 1 | 1 | 3 | 1 | 1 | 1 | 3 | 1 | 1 | 1 | 1 | 1 |
|  | Week 5 | 1 | 1 | 1 | 1 | 1 | 1 | 1 | 1 | 2 | 1 | 1 | 1 | 1 | 1 |
|  | Week 6 | 1 | 1 | 1 | 1 | 1 | 1 | 1 | 1 | 1 | 1 | 1 | 1 | 1 | 1 |
| Patient 3 | Week 1 | 1 | 1 | 1 | 1 | 3 | 1 | 1 | 1 | 2 | 1 | 1 | 1 | 2 | 1 |
|  | Week 2 | 1 | 2 | 1 | 1 | 3 | 4 | 2 | 1 | 2 | 1 | 1 | 2 | 4 | 4 |
|  | Week 3 | NA | NA | NA | NA | NA | NA | NA | NA | NA | NA | NA | NA | NA | NA |
|  | Week 4 | 1 | 1 | 2 | 1 | 4 | 4 | 2 | 1 | 2 | 1 | 1 | 1 | 1 | 1 |
|  | Week 5 | NA | NA | NA | NA | NA | NA | NA | NA | NA | NA | NA | NA | NA | NA |
|  | Week 6 | 1 | 1 | 1 | 1 | 2 | 2 | 2 | 1 | 2 | 1 | 1 | 1 | 1 | 1 |
| Patient 4 | Week 1 | 1 | 1 | 1 | 2 | 3 | 5 | 6 | 2 | 4 | 1 | 1 | 1 | 1 | 1 |
|  | Week 2 | 1 | 4 | 1 | 1 | 3 | 3 | 3 | 1 | 3 | 1 | 1 | 1 | 1 | 1 |
|  | Week 3 | 1 | 2 | 1 | 1 | 2 | 2 | 2 | 1 | 2 | 1 | 1 | 1 | 1 | 1 |
|  | Week 4 | 1 | 3 | 2 | 1 | 2 | 2 | 2 | 1 | 2 | 1 | 1 | 1 | 1 | 1 |
|  | Week 5 | 1 | 8 | 8 | 1 | 8 | 1 | 1 | 1 | 1 | 1 | 1 | 1 | 1 | 1 |
|  | Week 6 | 1 | 3 | 3 | 1 | 2 | 2 | 2 | 1 | 2 | 1 | 1 | 1 | 1 | 1 |
| Patient 5 - Cycle 1 | Week 1 | 2 | 1 | 1 | 1 | 1 | 1 | 1 | 1 | 1 | 1 | 1 | 1 | 1 | 1 |
|  | Week 2 | 1 | 1 | 1 | 1 | 1 | 1 | 1 | 1 | 1 | 1 | 1 | 1 | 1 | 1 |
|  | Week 3 | 1 | 1 | 1 | 1 | 1 | 1 | 1 | 1 | 1 | 1 | 1 | 1 | 1 | 1 |
|  | Week 4 | 1 | 1 | 1 | 1 | 1 | 1 | 1 | 1 | 1 | 1 | 1 | 1 | 1 | 1 |
|  | Week 5 | 1 | 1 | 1 | 1 | 1 | 1 | 1 | 1 | 1 | 1 | 1 | 1 | 1 | 1 |
|  | Week 6 | 1 | 1 | 1 | 1 | 1 | 1 | 1 | 1 | 1 | 1 | 1 | 1 | 1 | 1 |
| Patient 5 – Cycle 2 | Week 1 | 1 | 1 | 1 | 1 | 1 | 1 | 1 | 1 | 1 | 1 | 1 | 1 | 1 | 1 |
|  | Week 2 | 6 | 1 | 1 | 1 | 1 | 1 | 7 | 1 | 1 | 1 | 1 | 1 | 7 | 1 |
|  | Week 3 | 1 | 1 | 1 | 1 | 1 | 1 | 1 | 1 | 1 | 1 | 1 | 1 | 1 | 1 |
|  | Week 4 | 1 | 1 | 1 | 1 | 1 | 1 | 1 | 1 | 1 | 1 | 1 | 1 | 1 | 1 |
|  | Week 5 | NA | NA | NA | NA | NA | NA | NA | NA | NA | NA | NA | NA | NA | NA |
|  | Week 6 | NA | NA | NA | NA | NA | NA | NA | NA | NA | NA | NA | NA | NA | NA |
| Patient 6 – Cycle 1 | Week 1 | 1 | 1 | 1 | 1 | 6 | 1 | 1 | 1 | 3 | 1 | 1 | 1 | 1 | 1 |
|  | Week 2 | 1 | 1 | 1 | 1 | 2 | 1 | 1 | 1 | 2 | 1 | 1 | 1 | 1 | 1 |
|  | Week 3 | 1 | 1 | 1 | 1 | 1 | 1 | 1 | 1 | 2 | 1 | 1 | 1 | 1 | 3 |
|  | Week 4 | 1 | 1 | 1 | 1 | 1 | 3 | 1 | 1 | 1 | 1 | 1 | 1 | 1 | 3 |
|  | Week 5 | 1 | 1 | 1 | 1 | 1 | 1 | 2 | 1 | 1 | 1 | 1 | 1 | 1 | 1 |
|  | Week 6 | 1 | 1 | 1 | 1 | 1 | 1 | 2 | 1 | 1 | 1 | 1 | 1 | 1 | 1 |
| Patient 6 – Cycle 2 | Week 1 | 1 | 1 | 1 | 1 | 5 | 1 | 1 | 1 | 1 | 1 | 1 | 1 | 1 | 1 |
|  | Week 2 | 1 | 1 | 1 | 1 | 5 | 1 | 1 | 1 | 1 | 1 | 1 | 1 | 1 | 1 |
|  | Week 3 | 1 | 1 | 1 | 1 | 5 | 1 | 1 | 1 | 1 | 1 | 1 | 1 | 1 | 1 |
|  | Week 4 | 1 | 1 | 1 | 1 | 5 | 1 | 1 | 1 | 1 | 1 | 1 | 1 | 1 | 1 |
|  | Week 5 | 1 | 1 | 1 | 1 | 5 | 1 | 1 | 1 | 1 | 1 | 1 | 1 | 1 | 1 |
|  | Week 6 | 1 | 1 | 1 | 1 | 5 | 1 | 1 | 1 | 1 | 1 | 1 | 1 | 1 | 1 |
| Patient 7 | Week 1 | NA | NA | NA | NA | NA | NA | NA | NA | NA | NA | NA | NA | NA | NA |
|  | Week 2 | 1 | 1 | 1 | 1 | 1 | 7 | 1 | 1 | 7 | 1 | 1 | 1 | 1 | 1 |
|  | Week 3 | 1 | 1 | 1 | 1 | 1 | 1 | 1 | 1 | 1 | 1 | 1 | 1 | 1 | 1 |
|  | Week 4 | 1 | 1 | 1 | 1 | 1 | 1 | 1 | 1 | 1 | 1 | 1 | 1 | 1 | 1 |
|  | Week 5 | NA | NA | NA | NA | NA | NA | NA | NA | NA | NA | NA | NA | NA | NA |
|  | Week 6 | NA | NA | NA | NA | NA | NA | NA | NA | NA | NA | NA | NA | NA | NA |

*Note:* Adverse side effects reported based on the questionnaire of Thair et al. (2017). The questionnaire is completed by the clinician once a week. Values range from 1 (absent) to 10 (severe).; NA = not available. * Questions 3, 8, 10 and 11 are pseudo-items (i.e., control questions)
